# Combined performance of screening and variable selection methods in ultra-high dimensional data in predicting time-to-event outcomes

**DOI:** 10.1186/s41512-018-0043-4

**Published:** 2018-09-26

**Authors:** Lira Pi, Susan Halabi

**Affiliations:** Department of Biostatistics and Bioinformatics, Duke University Medical Center, Durham, NC 27710 USA

**Keywords:** Variable selection, Calibration, Overfitting, Machine learning, Proportional hazards model, Prognostic models, Elastic net, Random forest, High dimensional data, Germline single-nucleotide polymorphism, Survival outcomes

## Abstract

**Background:**

Building prognostic models of clinical outcomes is an increasingly important research task and will remain a vital area in genomic medicine. Prognostic models of clinical outcomes are usually built and validated utilizing variable selection methods and machine learning tools. The challenges, however, in ultra-high dimensional space are not only to reduce the dimensionality of the data, but also to retain the important variables which predict the outcome. Screening approaches, such as the sure independence screening (SIS), iterative SIS (ISIS), and principled SIS (PSIS), have been developed to overcome the challenge of high dimensionality. We are interested in identifying important single-nucleotide polymorphisms (SNPs) and integrating them into a validated prognostic model of overall survival in patients with metastatic prostate cancer. While the abovementioned variable selection approaches have theoretical justification in selecting SNPs, the comparison and the performance of these combined methods in predicting time-to-event outcomes have not been previously studied in ultra-high dimensional space with hundreds of thousands of variables.

**Methods:**

We conducted a series of simulations to compare the performance of different combinations of variable selection approaches and classification trees, such as the least absolute shrinkage and selection operator (LASSO), adaptive least absolute shrinkage and selection operator (ALASSO), and random survival forest (RSF), in ultra-high dimensional setting data for the purpose of developing prognostic models for a time-to-event outcome that is subject to censoring. The variable selection methods were evaluated for discrimination (Harrell’s concordance statistic), calibration, and overall performance. In addition, we applied these approaches to 498,081 SNPs from 623 Caucasian patients with prostate cancer.

**Results:**

When *n* = 300, ISIS-LASSO and ISIS-ALASSO chose all the informative variables which resulted in the highest Harrell’s c-index (> 0.80). On the other hand, with a small sample size (*n* = 150), ALASSO performed better than any other combinations as demonstrated by the highest c-index and/or overall performance, although there was evidence of overfitting. In analyzing the prostate cancer data, ISIS-ALASSO, SIS-LASSO, and SIS-ALASSO combinations achieved the highest discrimination with c-index of 0.67.

**Conclusions:**

Choosing the appropriate variable selection method for training a model is a critical step in developing a robust prognostic model. Based on the simulation studies, the effective use of ALASSO or a combination of methods, such as ISIS-LASSO and ISIS-ALASSO, allows both for the development of prognostic models with high predictive accuracy and a low risk of overfitting assuming moderate sample sizes.

## Background

The proportional hazards (PH) model [1, 2] has been widely used for predicting time-to-event outcomes. The partial likelihood estimation of the PH model, however, is not appropriate for exploring the simultaneous relationship of the high dimensional variables with outcomes. For this reason, variable selection approaches, such as the least absolute shrinkage and selection operator (LASSO) [3], adaptive LASSO (ALASSO) [4], and a specific extension of random survival forests (RSF) [5], have been widely used to select the informative variables in a high dimensional setting [6–9]. In addition, LASSO, ALASSO, and RSF have been extended to time-to-event endpoints that are subject to censoring. Although these methods are capable of reducing the number of variables in high dimensionality, Fan et al. [10] and Zhao and Li [11] proposed methods, such as the sure independence screening (SIS) [10], the iterative SIS (ISIS) [10], and the principled SIS (PSIS) [11], to expedite computing time and improve estimation accuracy in a ultra-high dimensional setting. Furthermore, Fan et al. define ultra-high dimensionality by the exponential growth of the dimensionality in the sample size (that is, log(*p*) = *O*(*n*^*a*^) for some *a* ∈ (0,0.5)) [9].

The SIS or PSIS aims to reduce the ultra-high dimensional space to a manageable subset which encompasses the important variables. We define important variables as those in which the true coefficients are not equal to zero marginally on the survival outcomes, assuming a linear association between the log-hazard function and the variables in the proportional hazard model. SIS or PSIS can select important variables, just as LASSO or ALASSO. When SIS or PSIS is combined with LASSO or ALASSO, however, SIS or PSIS is applied prior to running LASSO or ALASSO in the same way as employed in [9]. To emphasize the processing order among the methods, we broadly classify these methods into two groups: screening approaches which follow the genomic literature (SIS and PSIS) and variable selection methods (LASSO and ALASSO). Fan et al. [9, 10] theoretically proved that SIS is highly likely to choose all the important variables and showed that SIS and its variants are more efficient in excluding unimportant variables than LASSO alone.

We chose LASSO, ALASSO, and RSF due to their wide application in genomic medicine in selecting informative variables in high dimensional settings. Fan et al. [9, 10] showed that LASSO selects too many uninformative variables and thus the authors proposed to combine SIS with LASSO to efficiently exclude uninformative variables while keeping all the informative ones. Thus, the concept of implementing SIS, ISIS, and PSIS in high dimensional space before running ALASSO and RSF was innovative [9–11]. Moreover, statisticians who are not familiar with SIS, ISIS, and PSIS may want to consider using these variable methods as alternatives to select important variables and ultimately develop prognostic models with higher predictive accuracy in high dimensional space.

We are interested in identifying important germline single-nucleotide polymorphisms (SNPs) and integrating them into a validated prognostic model of overall survival in patients with metastatic prostate cancer. The data included 498,081 SNPs processed from blood samples from 623 Caucasian patients with prostate cancer after passing quality control. While the abovementioned variable selection approaches have theoretical justification in selecting important SNPs, the comparison and the performance of these combined methods have not been studied in ultra-high dimensional space with time-to-event endpoints. In other words, it is unknown if the combined variable selection methods would perform well in a context of an ultra-high number of variables. Our primary goal is to compare the performance of the methods in predicting a time-to-event outcome via a simulation study with 100,000 (100 k) variables. There is a plethora of variable selection methods, but it is not always apparent which approach is best to use in identifying important variables in ultra-high dimensional space. Thus, another purpose of this paper is to provide applied statisticians with a basic understanding of these combined methods and to make recommendations on how best to use them.

The rest of this paper is organized as follows. We provide an overview of the widely used variable selection methods in the “Methods” section while offering a concise discussion of their advantages and disadvantages. In the “Results” section, we describe the design of the simulation studies and the comparison among the four single variable selection methods and the nine combinations of the variable selection methods. An ultra-high dimensional example is used where the data are analyzed using a combination of methods to identify SNPs that will predict overall survival time for metastatic prostate cancer patients (“Discussion” section). The results of the simulations and recommendations are discussed in the “Conclusion” section.

## Methods

**Fig. 1:**
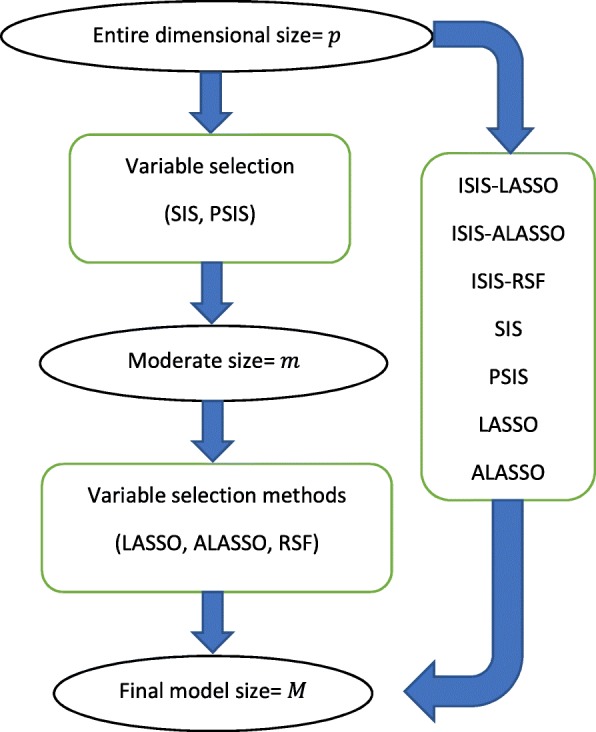
Overall diagram of the screening approaches and the variable selection methods

Let *p* be the number of variables. A linear association between the log-hazard function and the *p*-dimensional variables is assumed in the PH model as follows:

1 $$ h\left(t|x\right)={h}_0(t){e}^{\boldsymbol{x}\boldsymbol{\beta }} $$

where *h*(*t*| ***x***) is a hazard function of time-to-event *t*, provided that each of the *p* variables, ***x*** = (***x***_1_, ***x***_2_, …, ***x***_*p*_ )^′^, is a vector with sample size *n*, for the ***x***_*i*_ = (*x*_*i*1_, *x*_*i*2_, …, *x*_*in*_ )^′^ for *i* = 1, …, *p*. The parameters ***β*** = (*β*_1_, *β*_2_, …, *β*_*p*_)^′^ and *h*_0_(*t*) are an arbitrary parameter-free baseline hazard function. We assume that the important variables are associated with the time-to-event based on (1). We consider the variable selection methods in order to only choose important variables of clinical outcomes, as presented in Fig. 1.

### Screening approaches

**Fig. 2:**
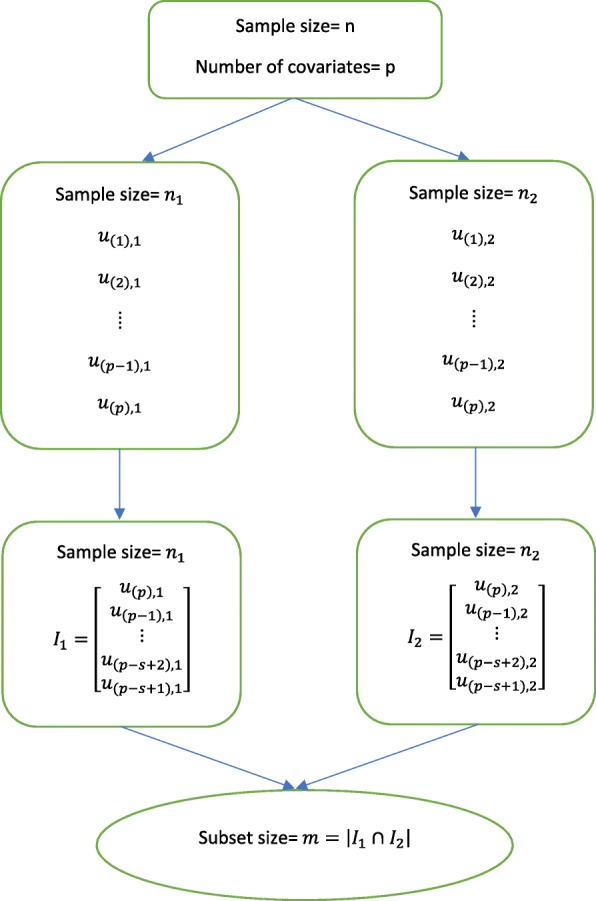
Process of the aggressive variant of sure independence screening (SIS)

We refer to SIS, ISIS, and PSIS as screening or variable selection approaches, and these words are used interchangeably. Fan et al. [10] introduced the SIS to decrease the number of variables *p* from the ultra-high to a reduced subset size *m* for time-to-event outcomes. They choose *m* as ≤⌊*n*/ log(*n*)⌋ or ⌊*n*/(4 log(*n*))⌋ depending on the different variants of SIS. The former is known as “aggressive” SIS, and the latter is named “vanilla” SIS. We consider the aggressive SIS since its performance is shown to be more effective in high dimensional setting [10]. The aggressive SIS starts with randomly splitting the sample into two partitions, *n*_1_ and *n*_2_, then SIS computes the marginal correlation between a single variable and the survival outcome within each partition. This is called marginal utility and is denoted as *u*_*k*, 1_ and *u*_*k*, 2_ for *k* = 1, …, *p*. The utility is obtained by maximizing the partial likelihood of each variable in the following way:

2 $$ {\displaystyle \begin{array}{c}{u}_{k,1}={\max}_{\beta_k}\left({\sum}_{i\in {n}_1}{\delta}_i{x}_{ik}{\beta}_k-{\sum}_{i\in {n}_1}{\delta}_i\log \left\{{\sum}_{j\in R\left({y}_i\right)}\exp \left({x}_{jk}{\beta}_k\right)\right\}\right),\\ {}{u}_{k,2}={\max}_{\beta_k}\left({\sum}_{i\in {n}_2}{\delta}_i{x}_{ik}{\beta}_k-{\sum}_{i\in {n}_2}{\delta}_i\log \left\{{\sum}_{j\in R\left({y}_i\right)}\exp \left({x}_{jk}{\beta}_k\right)\right\}\right),\end{array}} $$

in which *δ*_*i*_ is the censoring indicator, *x*_*ik*_ is the *k*th element among the *p* variables, and *R*(*y*_*i*_) is the risk set right before the time *y*_*i*_, i.e., *R*(*y*_*i*_) = {*j*; *y*_*j*_ ≥ *y*_*i*_}. By ordering all variables in increasing order of their corresponding marginal utilities, *u*_(1)_ < *u*_(2)_ < … < *u*_(*p* − 1)_ < *u*_(*p*)_, one selects the top *s* $\left(=\left\lfloor \frac{n}{\log (n)}\right\rfloor \right)$ ranked variables for each partition. The details are presented in Fig. 2. Let *I*_1_ and *I*_2_ be the index sets of the selected *s* variables and are expressed as

3 $$ s=\left|{I}_1=\left\{1\le k\le p;{u}_{k,1}\ge {\gamma}_{s,1}\right\}\right|=\left|{I}_2=\left\{1\le k\le p;{u}_{k,2}\ge {\gamma}_{s,2}\right\}\right|, $$

where *γ*_*s*, 1_ and *γ*_*s*, 2_ are the cutoff values depending on *s* for each partition. The asymptotic probability that *I*_1_ ∩ *I*_2_ contains all the important variables is 1. This is the reason why this method is called sure screening.

The SIS is, however, unable to select important variables that are weakly and marginally associated with right-censored outcomes. Furthermore, *I*_1_ ∩ *I*_2_ is most likely to retain unimportant variables that are highly correlated with the important variables [10]. To reduce the false negative and false positive rates, Fan et al. [10] also proposed ISIS and its aggressive variant. The iterative SIS begins similarly to SIS in the first iteration and derives the first subset, *O*_1_ containing variables selected from a variable selection method. Let *m*_1_ = |*O*_1_| denote the first subset size. The second iteration repeats SIS but with the (*p* − *m*_1_) variables and calculates the conditional utility for each partition given *m*_1_ variables. The selection method is again applied to the union set of *m* variables and *O*_1_ from which the second subset *O*_2_ is driven. The process runs iteratively until *O*_*j* − 1_ = *O*_*j*_, for some *j* which is the number of iterations or until a pre-specified number of iterations has been reached.

Despite the high probability for ISIS to select the important variables, there is no theoretical justification for choosing the cutoff values *γ*_*s*, 1_ and *γ*_*s*, 2_. The lack of theoretical explanation motivates Zhao and Li [11] to develop the principled SIS for time-to-event endpoints. Furthermore, Zhao and Li provide the mathematical reasoning for the cutoff value *γ*_*m*_ by using the standard normal distribution and hence controlling the false positive rate. The PSIS requires the maximum partial likelihood estimates (MPLE) of the coefficients to be written as:

4 $$ {\widehat{\beta}}_k=\underset{\beta_k}{\mathrm{argmax}}\left({\sum}_{i=1}^n{\delta}_i{x}_{ik}{\beta}_k-{\sum}_{i=1}^n{\delta}_i\log \left\{{\sum}_{j\in R\left({y}_i\right)}\exp \left({x}_{jk}{\beta}_k\right)\right\}\right). $$

Once the false positive rate *q*_*m*_ is chosen, the cutoff value *γ*_*m*_ will be the quantile of the standard normal distribution, i.e., *γ*_*m*_ = *Φ*^−1^(1 − *q*_*m*_/2), assuming that the asymptotic null distribution of ${I}_k{\left({\widehat{\beta}}_k\right)}^{1/2}{\widehat{\beta}}_k$ is *N*(0, 1), where *I*_*k*_ is the information matrix. Hence, the *m* variables are chosen in the following manner:

5 $$ m=\left|1\le k\le p;{I}_k{\left({\widehat{\beta}}_k\right)}^{\frac{1}{2}}|{\widehat{\beta}}_k|\ge {\varPhi}^{-1}\left(1-{q}_m/2\right)\ \right|. $$

### Variable selection methods

It is possible that the subset filtered by SIS or PSIS includes not only the important *p*^∗^ variables but also several of the unimportant (*p* − *p*^∗^) variables. To remove the falsely selected variables, we employ some of the most widely used penalized regression methods in current clinical research, such as the LASSO and the ALASSO. Tibshirani [3] proposed LASSO with the L1 penalty while taking the survival analysis framework into consideration. The efficient computational algorithm is conducted in Simon et al. [12] by maximizing a scaled log partial likelihood of the PH model. The penalized maximum likelihood estimator is given by

6 $$ \widehat{\beta}=\underset{\beta }{\mathrm{argmax}}\left[\frac{2}{n}l\left(\beta \right)-\lambda {P}_{\alpha}\left(W,\beta \right)\right], $$

where

7 $$ {P}_{\alpha}\left(W,\beta \right)=\alpha {\left\Vert W\beta \right\Vert}_1+\frac{1}{2}\left(1-\alpha \right){\left\Vert \beta \right\Vert}_2^2. $$

Note that the R library glmnet scales the log partial likelihood by a factor of 2/*n* for convenience. LASSO evaluates (6) with a scalar *α* = 1 and a matrix *W* = *I*_*p*_ in which *I*_*p*_ is the *p*-dimensional identity matrix in (7). ALASSO identically plugs in *α* = 1, but instead, a diagonal matrix, *W*, with $1/\mid \overset{\sim }{\beta}\mid$ as the diagonal entries in which $\overset{\sim }{\beta }={\left({\overset{\sim }{\beta}}_1,{\overset{\sim }{\beta}}_2,\dots, {\overset{\sim }{\beta}}_p\right)}^{\prime }$ is a vector containing the MPLE of the log partial likelihood *l*(*β*). The coefficient estimates are obtained corresponding to the value of *λ* that minimizes the mean cross-validated partial likelihood error in this paper. To be specific, 10-fold cross-validation was used. Only variables with non-zero coefficient estimates are finally selected by the penalized algorithm.

Unlike the penalized regression approaches, the random survival forest (RSF) is a non-parametric method and an extension of Breiman’s random forests [13] for analyzing right-censored outcomes. These methods are known for their ability to easily deal with nonlinear effects, correlated variables, and variable interactions [13]. We implement minimal depth thresholding, which stems from the idea of observing the most important variables that are located on the upper nodes in a tree. Ishwaran et al. [14] provide the null distribution of the minimal depth of a maximal subtree that is given by

8 $$ P\left({D}_v=D(S)|v\ \mathrm{is}\ \mathrm{noisy},{l}_0,\dots, {l}_{D(S)-1}\right)=1-{\sum}_{d=0}^{D(S)-1}{\left(1-\frac{1}{p}\right)}^{L_d}\left(1-{\left(1-\frac{1}{p}\right)}^{l_d}\right), $$

where *D*_*v*_ is the minimal depth for a given variable *v*, *D*(*S*) is the depth of the tree S, *l*_*d*_ is the number of nodes at depth *d*, and ${L}_d=\sum \limits_{i=0}^{d-1}{l}_i$. According to Ishwaran et al. [14], a variable *v* is chosen if its forest averaged minimal depth, *D*_*v*_ = *d*_*v*_, is less than or equal to the mean minimal depth of *D*_*v*_ under the null distribution of (8). Thus, we build final models with the variables meeting the (mean) minimal depth thresholding.

### Simulation studies

We conducted a series of simulations to compare the performance of the combined variable selection approaches in the ultra-high dimensional setting within the survival framework. We used single variable selection methods as references and assumed a linear relationship between the log-hazard function and the significant variables via the non-zero constant coefficients. In addition, we assumed that the baseline hazard function had a value of 1. We considered *p*^∗^ to be six because most articles in the context of the ultra-high dimensional data assumed six or less important variables in their simulation studies as these methods are computationally intensive [10, 15, 16]. Another reason for considering a small number of important variables is that many genome-wide association (GWAS) analysis identified only a few SNPs that are clinically important [17–20].

We limited *p* to 100 k, each variable having three classes since they were similar to the SNP genetic data that served as our motivation example. The random samples were generated assuming that the variables are mutually independent and identically distributed (IID). The IID variables were randomly and independently sampled from a multinomial distribution being specified with probabilities for the three classes, (0, 1, 2) as ((1 − *q*)^2^, 2*q*(1 − *q*), *q*^2^), respectively, which followed the Hardy-Weinberg equilibrium (HWE) [21, 22]. In the context of genetic data, *q* is the minor (risk) allele frequency (MAF) of a SNP and was assumed to represent 0.15 of SNPs. The MAF refers to the frequency of the allele with frequency no more than 50% in a population [22]. The three probabilities were formed given that the pairs of alleles were known to be independent, and hence, there was no deviation from the HWE.

The true regression coefficient values ***β*** were randomly chosen from (−1)^*u*^(*a*+| *z*| ), where *u* was sampled from a Bernoulli distribution having parameter 0.5, *z* generated from the standard Gaussian distribution. We followed Fan et al. [9] and set *a* = 1 and *a* = 2 for weak and strong signal strength, respectively. We considered two scenarios for the true values of ***β.***

Strong signal:

$$ \boldsymbol{\beta} ={\left(2.527186,2.443898,2.152147,-2.388758,2.156502,-2.003314\right)}^{\prime }, $$

Weak signal:

$$ \boldsymbol{\beta} ={\left(1.036478,-1.073296,-1.250946,1.138729,-1.128361,1.145263\right)}^{\prime }. $$

The true coefficients were multipliers of the first six variables, and hence, the other 99,994 variables had zero coefficients. Failure times were assumed following a Weibull distribution. The hazard function *h*_*w*_(*t*| *x*) was the function of the variables from a Weibull distribution, and it is expressed as

9 $$ {h}_w\left(t|x\right)=\tau {t}^{\tau -1}\times \mathit{\exp}\left\{\tau \left({x}_1{\beta}_1+{x}_2{\beta}_2+{x}_3{\beta}_3+{x}_4{\beta}_4+{x}_5{\beta}_5+{x}_6{\beta}_6\right)\right\}, $$

where *τ* was a shape parameter of the Weibull distribution. The failure times were randomly drawn from the Weibull distribution assuming *τ*=1.5. Also, we set the inverse of exp{*τ*(*x*_1_*β*_1_ + *x*_2_*β*_2_ + *x*_3_*β*_3_ + *x*_4_*β*_4_ + *x*_5_*β*_5_ + *x*_6_*β*_6_)} as the scale parameter.

The censoring rate *C* was specified as 20% across the simulation studies. Censored times were assumed to follow a uniform distribution on [0, *θ*_*c*_]. For each specific censoring rate, *θ*_*c*_ was determined using the Newton-Raphson iteration (Appendix (A3) and (A5) of [23]). The failure time, *T*_*i*_, for *i* = 1, …, *n*, was randomly generated from (9), whereas the censoring times *C*_*i*_, for *i* = 1, …, *n* was randomly sampled from uniform distribution. Then, the observed failure times and censoring indicators were *t*_*i*_ = min(*T*_*i*_, *C*_*i*_) and *δ*_*i*_ = *I*(*T*_*i*_ ≤ *C*_*i*_), respectively. We considered sample sizes of 150 and 300.

Because of the intensive computing time (100 k variables), we generated 300 data sets for each simulation scenario. Single variable selection approaches (SIS, PSIS, LASSO, and ALASSO) were examined as references. In addition, nine combinations of the variable selection methods were considered. These are ISIS-LASSO, SIS-LASSO, PSIS-LASSO, ISIS-ALASSO, SIS-ALASSO, PSIS-ALASSO, ISIS-RSF, SIS-RSF, and PSIS-RSF. It is important to note that it is not possible to run ISIS alone as it works simultaneously with SIS in combination with a variable selection method. We sought to run RSF by itself; however, it had insufficient memory. We used the default maximum number of iteration (five) from the SIS library in R version 0.6 when running the ISIS combinations. To expedite computing time in running RSF, we set the number of trees in the forest and the maximum number of random split points to be 100 and 10, respectively, to increase computing speed. The split points split for a variable. For PSIS, we set the false positive rate at 0.001 (=*q*_*m*_ from (5)) over all simulation data sets as this will limit PSIS from selecting hundreds of variables in a reduced subset.

Prognostic models were developed based on the training datasets, whereas independent testing sets were generated from the underlying joint distribution of the variables. The testing sets had much larger sample sizes (*n* = 2000) than the training sets, as suggested by Hothorn et al. [24]. The above variable selection approaches were compared for their predictive ability using overall performance, discrimination, and calibration. For the overall performance, we picked up Graf et al. ${R}_{BS}^2$ [25], which assesses the percentage gain in predictive accuracy compared to the null model (model with no variables) at a single time point. In this paper, ${R}_{BS}^2$ was evaluated at 2 years because it was very close to the median overall survival time in our motivation example of prostate cancer. A positive value of ${R}_{BS}^2$ means that a prognostic model predicts better relative to the null model, whereas a negative value indicates that the model predicts poorly relative to the null model. Discrimination describes the ability of a prognostic model to distinguish between patients with and patients without the outcome of interest [26]. Discrimination was measured using Harrell’s c-index. A value of 0.5 represents random prediction whereas a value of 1 represents perfect discrimination [26]. Calibration refers to the agreement between the observed survival probability at 2 years versus the predicted probability at 2 years. Patients were divided into quartiles, and the average predicted survival probability was compared with the observed survival probability similar to how it is described by Harrell [26]. The calibration slope was estimated by regressing new survival outcomes on the predicted prognostic index [27]. Lastly, overfitting was evaluated by using the rms library in R using the validated command with 100 bootstrapped samples generated from the original sample.

The high computational burden was alleviated by using shared cluster computers at the University of Iowa Supercomputer Center. The LASSO, ALASSO, and RSF were executed in R using glmnet library version 2.0-5 and randomForestSRC version 2.1.0. We created R codes for PSIS according to the algorithms in [9] because there is no available library for PSIS. We employed R codes from the SIS library (version 0.6) and combined it with ALASSO and RSF in accordance with the techniques described in [28] since SIS can only implement SIS-LASSO or ISIS-LASSO. The R codes were written by the first author and are available on https://duke.box.com/s/q2wj7m4gxv0gnslaw91nooindjjat6cf.

## Results

### Overall performance

Table 1 shows the means and standard deviations of ${R}_{BS}^2$ values evaluated at 2 years out of the 300 simulations for the training sets. The highest ${R}_{BS}^2(2)$ were observed for PSIS-LASSO and PSIS-ALASSO when *n* = 150 regardless of the signal strength. For *n* = 300, and assuming a weak signal among the six variables, LASSO had the highest ${R}_{BS}^2(2)$ which was 83% (Table 1). This implies that the final model containing the selected variables from LASSO had 83% gain relative to a null model in explaining the survival outcomes at 2 years. When *n* = 300, and assuming a strong signal among the six variables, PSIS-LASSO and PSIS had ${R}_{BS}^2(2)$ above 85%, whereas LASSO had negative average ${R}_{BS}^2(2)$, indicating that the average model size of 108 variables performed poorly compared to the null model.

**Table 1:** Mean (SD) of ${R}_{BS}^2(2)$ for the training sets over 300 simulations

Selection approach	Weak signal		Strong signal	
	*n* = 150	*n* = 300	*n* = 150	*n* = 300
ISIS-LASSO	0.122 (0.109)	0.578 (0.071)	0.335 (0.318)	0.797 (0.071)
ISIS-ALASSO	0.108 (0.108)	0.578 (0.071)	0.337 (0.322)	0.797 (0.071)
ISIS-RSF	0.069 (0.088)	0.564 (0.105)	0.232 (0.284)	0.790 (0.086)
SIS	0.065 (0.085)	0.303 (0.116)	0.114 (0.119)	0.491 (0.177)
SIS-LASSO	0.112 (0.093)	0.306 (0.113)	0.142 (0.119)	0.491 (0.177)
SIS-ALASSO	0.093 (0.092)	0.306 (0.113)	0.137 (0.120)	0.491 (0.177)
SIS-RSF	0.063 (0.082)	0.294 (0.125)	0.097 (0.104)	0.482 (0.178)
PSIS	0.756 (0.116)	0.811 (0.038)	0.642 (0.183)	0.869 (0.047)
PSIS-LASSO	0.875 (0.051)	0.805 (0.037)	0.869 (0.062)	0.874 (0.049)
PSIS-ALASSO	0.874 (0.052)	0.780 (0.042)	0.869 (0.063)	0.849 (0.057)
PSIS-RSF	0.688 (0.088)	0.413 (0.109)	0.655 (0.111)	0.555 (0.167)
LASSO	0.758 (0.138)	0.834 (0.043)	0.787 (0.136)	− 0.211 (0.036)
ALASSO	0.583 (0.115)	0.611 (0.058)	0.812 (0.095)	0.815 (0.063)

*SD* standard deviation

We present the mean and standard deviation (SD) of the final model sizes in Table 2. We observed that PSIS, PSIS-LASSO, and PSIS-ALASSO chose an excessive number of uninformative variables given that the number of informative variables was fixed at six. When *n* = 300, and assuming a strong signal among the six variables, the prediction models that employed LASSO performed poorly compared to the null model. When the sample size was 300 and a strong signal was assumed, the combinations with ISIS chose a small number of variables and their ${R}_{BS}^2(2)$ values were almost 80% (Table 2).

**Table 2:** Mean (SD) of final model size for the training sets over 300 simulations

Selection approach	Weak signal		Strong signal	
	*n* = 150	*n* = 300	*n* = 150	*n* = 300
ISIS-LASSO	1.087 (1.130)	6.010 (0.672)	2.567 (2.265)	6.073 (0.261)
ISIS-ALASSO	0.993 (0.925)	5.943 (0.732)	2.397 (2.164)	6.033 (0.180)
ISIS-RSF	2.463 (0.550)	6.007 (0.954)	3.367 (1.777)	6.033 (0.304)
SIS	2.277 (0.448)	3.120 (0.925)	2.477 (0.500)	4.380 (0.955)
SIS-LASSO	0.773 (0.955)	3.033 (1.014)	1.217 (0.945)	4.377 (0.951)
SIS-ALASSO	0.750 (0.737)	3.003 (1.049)	1.100 (0.824)	4.373 (0.951)
SIS-RSF	2.057 (0.644)	3.033 (1.037)	2.240 (0.719)	4.327 (0.961)
PSIS	132.183 (11.557)	120.440 (10.707)	129.980 (11.203)	119.103 (10.702)
PSIS-LASSO	69.850 (7.238)	78.503 (7.301)	63.170 (7.212)	65.473 (6.786)
PSIS-ALASSO	52.613 (6.344)	49.007 (7.881)	42.487 (8.784)	32.067 (9.986)
PSIS-RSF	44.26 (10.512)	16.033 (5.713)	42.667 (11.009)	15.113 (5.159)
LASSO	26.619 (11.320)	61.437 (13.310)	58.347 (10.184)	108.470 (14.364)
ALASSO	19.948 (7.318)	45.010 (8.276)	33.793 (7.738)	67.147 (9.422)

*SD* standard deviation

Table 3 presents the mean number of important variables chosen in the final models over the 300 simulations in the training sets. In addition, we present the proportion of the number of unimportant variables selected in each model relative to the size of the final models. When *n* = 150, PSIS, PSIS-LASSO, PSIS-ALASSO, LASSO, and ALASSO selected four or more important variables on average. These final models chose too many unimportant variables as the proportions of unimportant variables were greater than 85% (Table 3). On the other hand, when *n* = 300 and the signal strength was weak, ISIS-LASSO, ISIS-ALASSO, and ISIS-RSF selected five or more important variables on average. These combinations however chose a small number of unimportant variables as the largest proportion was only 3.3% for ISIS-RSF (Table 3).

**Table 3:** Mean of the number of informative features (% of uninformative features) selected in final model over 300 simulations in the training sets

Selection approach	Weak signal		Strong signal	
	*n* = 150	*n* = 300	*n* = 150	*n* = 300
ISIS-LASSO	0.373 (64.1%)	5.913 (1.6%)	1.920 (35.3%)	6.000 (1.0%)
ISIS-ALASSO	0.383 (59.9%)	5.897 (0.9%)	1.983 (30.0%)	6.000 (0.5%)
ISIS-RSF	0.343 (88.3%)	5.817 (3.3%)	1.657 (61.1%)	5.973 (0.9%)
SIS	1.973 (89.4%)	0.160 (5.5%)	1.720 (70.7%)	0.023 (0.4%)
SIS-LASSO	0.303 (56.4%)	2.960 (2.8%)	0.757 (33.4%)	4.357 (0.4%)
SIS-ALASSO	0.303 (57.3%)	2.960 (1.6%)	0.757 (29.6%)	4.357 (0.3%)
SIS-RSF	0.293 (88.8%)	2.880 (5.3%)	0.683 (69.4%)	4.317 (0.2%)
PSIS	4.577 (96.5%)	5.943 (95.0%)	5.437 (95.8%)	5.987 (94.9%)
PSIS-LASSO	4.547 (93.4%)	5.943 (92.4%)	5.437 (91.3%)	5.987 (90.8%)
PSIS-ALASSO	4.180 (91.9%)	5.943 (87.5%)	5.403 (86.5%)	5.987 (78.7%)
PSIS-RSF	2.183 (94.8%)	3.717 (73.8%)	3.703 (90.7%)	4.973 (63.3%)
LASSO	4.671 (80.0%)	6.000 (89.7%)	5.990 (89.4%)	6.000 (94.4%)
ALASSO	5.731 (65.0%)	6.000 (86.2%)	6.000 (81.1%)	6.000 (90.9%)

### Overfitting and discrimination

We also assessed overfitting using the original samples (training sets). Table 4 presents the mean optimism and corrected c-index over 100 boostrapped samples when the sample size is 300. We observed the lowest mean optimism with SIS combination when the signal was weak, but these approaches led to the smallest corrected c-index. On the other hand, PSIS-LASSO had the highest mean optimism. LASSO, ALASSO, and the ISIS combinations had reasonable c-indexes relative to the mean optimism (Table 4). The same pattern was observed when a strong signal among the important variables was assumed.

**Table 4:** Mean optimism and corrected c-index over 100 boostrapped samples

	Weak signal (*n* = 300)		Strong signal (*n* = 300)	
	Optimism	C-index corrected	Optimism	C-index corrected
ISIS-LASSO	0.010	0.654	0.005	0.794
ISIS-ALASSO	0.010	0.655	0.005	0.794
ISIS-RSF	0.010	0.641	0.005	0.791
SIS	0.008	0.426	0.007	0.627
SIS-LASSO	0.007	0.431	0.007	0.627
SIS-ALASSO	0.006	0.431	0.007	0.627
SIS-RSF	0.008	0.416	0.007	0.624
PSIS	*	*	0.119	0.770
PSIS-LASSO	0.082	0.745	0.043	0.836
PSIS-ALASSO	0.042	0.772	0.019	0.844
PSIS-RSF	0.026	0.574	0.016	0.721
LASSO	0.045	0.808	0.048	0.903
ALASSO	0.057	0.648	0.058	0.781

*Did not converge

The mean and standard deviation values of *C*_*H*_ over the 300 simulations in the testing sets are presented in Table 5. We observed that when the signal is weak and *n* = 150, ALASSO had the highest c-index. On the other hand, prediction models from ISIS-LASSO, ISIS-ALASSO, ISIS-RSF, and ALASSO had the highest average c-index when the sample size was 300 and the signal was weak. When the sample size was 150 and a strong signal was assumed, both LASSO and ALASSO had the highest c-index (Table 5). In contrast, when the sample size was large and a strong signal was assumed, all variable selection approaches had high c-indices. The ISIS combinations had the highest c-index, followed by LASSO and ALASSO-PSIS combinations, respectively.

**Table 5:** Mean (SD) of *C*_*H*_ for the testing sets over 300 simulations

Selection approach	Weak signal		Strong signal	
	*n* = 2000	*n* = 2000	*n* = 2000	*n* = 2000
ISIS-LASSO	0.532 (0.053)	0.820 (0.033)	0.644 (0.149)	0.895 (0.003)
ISIS-ALASSO	0.534 (0.054)	0.819 (0.037)	0.648 (0.152)	0.895 (0.003)
ISIS-RSF	0.531 (0.050)	0.815 (0.044)	0.630 (0.135)	0.894 (0.010)
SIS	0.528 (0.045)	0.696 (0.052)	0.575 (0.068)	0.805 (0.050)
SIS-LASSO	0.528 (0.045)	0.696 (0.052)	0.575 (0.068)	0.805 (0.050)
SIS-ALASSO	0.528 (0.045)	0.696 (0.052)	0.575 (0.068)	0.805 (0.050)
SIS-RSF	0.528 (0.044)	0.692 (0.057)	0.570 (0.063)	0.803 (0.051)
PSIS	0.527 (0.035)	0.711 (0.027)	0.590 (0.066)	0.856 (0.018)
PSIS-LASSO	0.608 (0.050)	0.737 (0.025)	0.769 (0.072)	0.873 (0.014)
PSIS-ALASSO	0.600 (0.048)	0.748 (0.027)	0.769 (0.085)	0.880 (0.014)
PSIS-RSF	0.556 (0.033)	0.696 (0.046)	0.665 (0.066)	0.832 (0.039)
LASSO	0.692 (0.072)	0.786 (0.011)	0.857 (0.018)	0.881 (0.005)
ALASSO	0.797 (0.071)	0.818 (0.006)	0.886 (0.005)	0.886 (0.004)

*SD* standard deviation

### Calibration

The mean and standard deviation of the calibration slopes for the testing sets are displayed in Table 6. Calibration slope identifies a linear association between the observed survival outcomes and the predicted prognostic index. When *n* = 300, the average calibration slopes from the prognostic models containing variables selected by ISIS-LASSO, ISIS-ALASSO, and ISIS-RSF were close to a value of 1. This implies that these prognostic models were well calibrated. On the other hand, when *n* = 150, most variable selection approaches had calibration slopes considerably below 1, suggesting overfitting.

**Table 6:** Mean (SD) of calibration slope for testing set over 300 simulations

Selection approach	Weak signal		Strong signal	
	*n* = 2000	*n* = 4000	*n* = 2000	*n* = 4000
ISIS-LASSO	0.345 (0.340)	0.944 (0.065)	0.646 (0.347)	0.965 (0.058)
ISIS-ALASSO	0.318 (0.343)	0.945 (0.065)	0.628 (0.373)	0.965 (0.058)
ISIS-RSF	0.205 (0.379)	0.936 (0.095)	0.486 (0.515)	0.965 (0.059)
SIS	0.210 (0.395)	0.887 (0.126)	0.433 (0.512)	0.955 (0.076)
SIS-LASSO	0.391 (0.336)	0.895 (0.091)	0.624 (0.326)	0.955 (0.076)
SIS-ALASSO	0.321 (0.373)	0.897 (0.091)	0.611 (0.339)	0.955 (0.076)
SIS-RSF	0.092 (2.247)	0.881 (0.129)	0.448 (0.583)	0.950 (0.080)
PSIS	0.004 (0.006)	0.245 (0.055)	0.013 (0.017)	0.370 (0.060)
PSIS-LASSO	0.077 (0.049)	0.355 (0.060)	0.200 (0.072)	0.530 (0.056)
PSIS-ALASSO	0.075 (0.046)	0.376 (0.072)	0.246 (0.116)	0.605 (0.100)
PSIS-RSF	0.078 (0.055)	0.574 (0.135)	0.241 (0.114)	0.806 (0.097)
LASSO	0.359 (0.126)	0.381 (0.076)	0.213 (0.086)	0.219 (0.055)
ALASSO	0.780 (0.131)	0.732 (0.069)	0.642 (0.115)	0.653 (0.069)

*SD* standard deviation

**Fig. 3:**
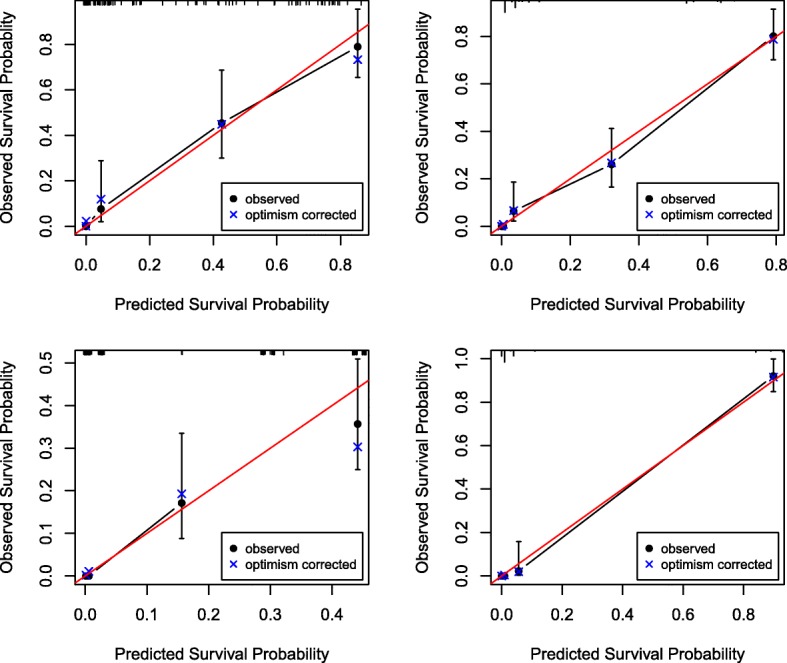
Calibration plots on training set for observed survival probability at 2 years versus predicted survival for **a** ALASSO with *n* = 150 and weak signal strength, **b** ISIS-ALASSO with *n* = 150 and weak signal strength, **c** ISIS-LASSO when *n* = 150 and strong signal strength, and **d** ISIS-LASSO when *n* = 300 and strong signal strength

The models were also evaluated for their calibration by plotting the observed versus the predicted probabilities for the variable selection approaches that had the calibration slopes closet to 1. Figure 3a–d presents the calibration plots of the observed survival probability versus the predicted probability at 2 years. The observed survival probability was close to the predicted probability when *n* = 150 and the signal strength is weak (Fig. 3a). We detected similar patterns as the observed survival probability was close to the predicted probability for ISIS-ALASSO when the sample size was 300 with a weak signal (Fig. 3b), for ISIS-LASSO when a strong signal among the variables is assumed and *n* = 150 (Fig. 3c) and for ISIS-LASSO when a strong signal among the variables is assumed *n* = 300 (Fig. 3d).

### Real-data example

The variable selection methods (SIS, PSIS, LASSO, LASSO, RSF) and the nine combinations were used to identify SNPs that predict overall survival in men with metastatic prostate cancer who participated in a phase III clinical trial (CALGB 90401). Overall survival was defined as the date of death from date of random assignment. We confined our analysis to 623 genetically defined Caucasians men who participated in the GWAS study [29]. Deaths were observed in 94% of the 623 Caucasian men.

Among all the SNPs, 498,081 SNPs were selected after quality control utilizing GenABEL (R package): call rate < 99%, *p* value of HWE < 1e−08, MAF < 0.05, and removal of non-autosomal SNPs. The missing SNP values were simply replaced with the average of non-missing SNP values. Although this may not be the optimal method for handling missing data, it is an effective ad hoc approach when the proportion of missing SNPs is relatively low. Eighty-five percent of the 498,081 SNPs that passed quality control were complete. The proportion of missing SNPs among 498,081 were 11.9% in 1 SNP, 2% had 2 SNPs, 0.5% had 3 SNPs, 0.2% had 4 SNPs missing, 0.10% had 5 SNPs missing, and 0.4% had more than 5 SNPs missing.

The dataset was randomly split into a 2:1 allocation ratio to training (*n* = 419) and testing (*n* = 204) sets, respectively. From the training set, important SNPs were selected using the different variable selection approaches. In all the proportional hazards models, SNPs were considered assuming additive models and were adjusted for risk score based on the predicted survival probability [30]. As expected, there was a slight indication of overfitting in the training set when the c-index from the original sample was compared to the bootstrapped samples. The corrected c-index is presented in Table 7. Three variable selection methods (ISIS-ALASSO, SIS-LASSO, and SIS-ALASSO) selected only the validated risk score. It is worth mentioning that for PSIS, PSIS-RSF, and LASSO, the corrected c-index could not be estimated due to the singularity problem among the predictors. The estimated coefficients of the selected SNPs from the training set were applied to the testing set, and we calculated the c-indices and the 95% confidence intervals. As presented in Table 7, ISIS-ALASSO, SIS-LASSO, and SIS-ALASSO combinations achieved the highest c-index of 0.67.

**Table 7:** c-indices based on the training and testing sets for the real example

Selection approach	No of SNPs selected	Training set (419)		Testing set (*n* = 204)
		Original c-index	Corrected c-index*	c-index* (95% CI)
ISIS-LASSO	2	0.649	0.646	0.664 (0.621–0.707)
ISIS-ALASSO	0	0.649	0.650	0.671 (0.624–0.719)
ISIS-RSF	2	0.650	0.645	0.669 (0.618–0.720)
SIS	2	0.650	0.646	0.669 (0.624–0.714)
SIS-LASSO	0	0.649	0.649	0.671 (0.626–0.717)
SIS-ALASSO	0	0.649	0.650	0.671 (0.620–0.723)
SIS-RSF	2	0.650	0.648	0.669 (0.623–0.716)
PSIS	40	0.749	–	0.572 (0.527–0.617)
PSIS-LASSO	28	0.746	0.727	0.568 (0.524–0.613)
PSIS-ALASSO	24	0.744	0.727	0.573 (0.528–0.617)
PSIS-RSF	35	0.748	–	0.575 (0.529–0.622)
LASSO	16	0.716	–	0.586 (0.540–0.632)
ALASSO	13	0.653	0.634	0.647 (0.601–0.693)

*Based on 200 bootstrapped samples

## Discussion

Advancement in laboratory technologies has led to ultra-high dimensional data that are now being routinely captured in clinical studies. A critical element of genomic medicine is implementing validated prognostic models for identifying patients with cancer for clinical trial design and/or for optimal therapy. For example, the Decipher signature has been developed for predicting metastasis after radical prostatectomy in men with prostate cancer [31–35]. The Decipher score was developed using random forest and has been independently validated [32–35]. Another example is the well-known oncotypeDx recurrence score that has been utilized to stratify randomization and to guide treatment in breast cancer patients in the TAILORx trial [36–38].

Prognostic models are usually built and validated utilizing common statistical methods and machine learning tools. The challenge in ultra-high dimensional space is not only to reduce the dimensionality of the data, but to keep the important molecular variables in predicting the time-to-event endpoints. Another major challenge which is common in high dimensional data is overfitting, which is identified by the high accuracy for a prognostic model based on a training set, but a low accuracy is observed when the model is evaluated on an external validation dataset.

This article explores the feasibility of combining several variable selection methods in an ultra-high dimensional setting for the purpose of developing prognostic models for time-to-event outcomes. To our knowledge, this is among the first articles that systematically compared the performance of ultra-high dimensional screening with variable selection methods for time-to-event endpoints.

When the sample size was small (*n* = 150) and the signal strength among the variables was weak, ALASSO outperformed all other approaches having the highest c-index and the calibration slopes closest to 1. Assuming a moderate sample size (*n* = 300) and a strong signal among the six variables, the combinations with ISIS not only selected all the important variables, but also excluded the unimportant variables. When *n* = 300 and the signal strength is weak, the combinations with ISIS had similar discriminative abilities compared with ALASSO in terms of high average c-index. The calibration slope, however, for ALASSO (0.732) indicated overfitting as compared with the slope value for ISIS-ALASSO (0.945). On the other hand, when *n* = 150 with a strong signal strength, ALASSO had the best performance. While the combinations with ISIS or SIS failed to select all the important variables, the combinations with PSIS chose too many unimportant variables.

Our results do not agree with Fan et al. [10], who demonstrated that SIS combined with LASSO is efficient in excluding unimportant variables rather than using only LASSO. This could be due to the fact that different performance measures (median and squared estimation errors) were reported [10]. Another possible explanation may be due to the fact that Fan et al. simulated correlated variables whereas we did not.

Other variable selection methods based on *p* values have also been used. One of these approaches is based on the false discovery rate (FDR). We have previously demonstrated the feasibility of the sequential use of FDR as a screening method with ISIS and other variable selection methods for predicting binary endpoints [39].

Turning back to our motivating example, the results from the prostate cancer data demonstrate the difficulty in choosing the important SNPs in predicting overall survival. The top three combinations, ISIS-ALASSO, SIS-LASSO, and SIS-ALASSO, outperformed other approaches, but they only selected the validated risk score [29]. The other combinations of variable selection methods had smaller c-indices even if they included more SNPs in addition to the validated risk score. This highlights the importance of validating the selected SNPs. Although external validation is considered the gold standard, model developers may not have access to external data. Other validation procedures, such as bootstrapping, are acceptable approaches [40, 41].

There are some caveats in applying the results from our simulation studies to real data. First, our simulations start with randomly generating categorical variables of which each has an identical probability to select three classes: 0, 1, and 2. The identical probability assumption, however, may not hold in a real problem. The second limitation is due to the independently and identically distributed assumption in generating the variables. Actual SNPs may be correlated with each other, although in our example, we observed zero correlation among SNPs across genes and modest correlations among SNPs within the same genes. Thirdly, we fixed several parameters in the simulations, such as the number of important variables, sample size, MAF, and signal strength, and all of these factors may have an impact on the simulation studies. Lastly, we used the R codes of the COXvarISISscad function in SIS (version 0.6) which was available at the time of the simulation study to combine it with ALASSO and RSF. The results from the aggressive variant of ISIS-LASSO using the latest SIS library (version 0.8-6) may produce different results when the sample size is small and the signal strength is weak. Despite the above limitations, these simulations are valuable to modelers.

Another vital point to consider is the computational time for the combination variable selection methods as it depends on the sample size. For instance, running ISIS-ALASSO and ALASSO for one training set with a sample size of 150 took 16 and 3 min, respectively. When *n* = 300, the processing time increased to 41 and 7 min for ISIS-ALASSO and ALASSO, respectively. Furthermore, running 500,000 variables over 200 bootstrapped samples was computationally intensive, and for the ISIS-LASSO combination, it took 6 h simply to choose the important variables. Although ISIS is computationally intensive, the combination performed well as indicated in our results.

Based on our simulations, we provide the following guidelines as a trade-off between predictive accuracy, calibration, and overfitting:

- When the sample size is small, and regardless of the strength of the signal among the variables, ALASSO performs well in selecting the important variables and achieving a high c-index.
- When the sample size is small and the signal among the covariate is strong, ALASSO again performs well in terms of having high c-index and high calibration slopes.
- When the sample size is large and a weak signal among the variables is present, ALASSO and the ISIS combinations had the highest performance and were comparable. The computing time, however, is much faster for ALASSO (six times faster than ISIS combination).
- When sample size is large and a strong signal is present, the ISIS combinations are the preferred approaches.

## Conclusion

Building and validating prognostic models of clinical outcomes will remain an important research area in 21st genomic medicine. Choosing the appropriate variable selection method for training a model is a critical step in developing a robust prognostic model. Based on the simulation studies, the effective use of ALASSO or a combination of methods, such as ISIS-LASSO and ISIS-ALASSO, allows both for the development of prognostic models with high predictive accuracy and a low risk of overfitting assuming moderate sample sizes.

## Abbreviations

ALASSO: Adaptive LASSO
AR: Auto-correlation
GWAS: Genome-wide association study
IID: Independent and identically distributed
ISIS: Iterative SIS
LASSO: Least absolute shrinkage and selection operator
PSIS: Principled SIS
RSF: Random survival forests
SIS: Sure independence screening
SNP: Single-nucleotide polymorphism
